# Smart Responsive and Controlled-Release Hydrogels for Chronic Wound Treatment

**DOI:** 10.3390/pharmaceutics15122735

**Published:** 2023-12-06

**Authors:** Xintao Jia, Zixuan Dou, Ying Zhang, Fanqin Li, Bin Xing, Zheming Hu, Xin Li, Zhongyan Liu, Wenzhuo Yang, Zhidong Liu

**Affiliations:** 1 State Key Laboratory of Component-Based Chinese Medicine, Tianjin University of Traditional Chinese Medicine, Tianjin 301617, China; xintaojia101@163.com (X.J.); zixuandou9509@163.com (Z.D.); zhangying120@hotmail.com (Y.Z.); xingbin1237@163.com (B.X.); 18624084757@163.com (Z.H.); xinli0026lx@163.com (X.L.); lzy1337518827@163.com (Z.L.); 18502240914@163.com (W.Y.); 2 Engineering Research Center of Modern Chinese Medicine Discovery and Preparation Technique, Ministry of Education, Tianjin University of Traditional Chinese Medicine, Tianjin 301617, China; 3 Haihe Laboratory of Modern Chinese Medicine, Tianjin 301617, China; 4 School of Traditional Chinese Medicine, Tianjin University of Traditional Chinese Medicine, Tianjin 301617, China; lfqtcm@163.com

**Keywords:** smart responsive hydrogel, chronic wound healing, drug delivery system, stimuli-responsive material, wound environment modulation

## Abstract

Chronic wounds are a major health challenge that require new treatment strategies. Hydrogels are promising drug delivery systems for chronic wound healing because of their biocompatibility, hydration, and flexibility. However, conventional hydrogels cannot adapt to the dynamic and complex wound environment, which involves low pH, high levels of reactive oxygen species, and specific enzyme expression. Therefore, smart responsive hydrogels that can sense and respond to these stimuli are needed. Crucially, smart responsive hydrogels can modulate drug release and eliminate pathological factors by changing their properties or structures in response to internal or external stimuli, such as pH, enzymes, light, and electricity. These stimuli can also be used to trigger antibacterial responses, angiogenesis, and cell proliferation to enhance wound healing. In this review, we introduce the synthesis and principles of smart responsive hydrogels, describe their design and applications for chronic wound healing, and discuss their future development directions. We hope that this review will inspire the development of smart responsive hydrogels for chronic wound healing.

## 1. Introduction

Chronic wounds do not heal normally over a long period and are usually a comorbidity of individuals with underlying diseases [1]. Chronic wounds include diabetic feet, venous legs, and pressure ulcers. In particular, the long healing time of chronic wounds imposes a huge health burden on patients and increases the healthcare costs of society [2,3].

Normally, wound healing occurs in four stages: haemostasis, inflammation, proliferation, and remodelling. These stages are linear and dynamic but overlap. However, chronic wounds remain in the inflammatory phase for an extended period. The main reasons for long-term non-healing are the prevention of angiogenesis, damage caused by reactive oxygen species (ROS), and bacterial infection [4,5,6]. Various treatment options for chronic wounds are available. Debridement is essential for good wound bed preparation and involves the removal of all necrotic and devitalised tissue that is incompatible with healing, as well as the removal of the surrounding callus. Sharp debridement is the simplest option, but enzymatic debridement and biosurgery offer more options for patients who reject anaesthesia or are concerned about pain. Oxygen therapy and negative pressure wound therapy are also tremendously helpful in wound healing; the former ameliorates the problems associated with the low oxygen supply arising from vascular disease, and the latter absorbs excess exudate. Wound offloading is an important adjunct therapy that relieves pressure and shear stress to promote healing and prevent wounds [7,8,9,10].

There is consensus in reports on the care of chronic wounds that the site should be maintained as moist, and wound dressings are the most widely used option to achieve such a humid environment [11,12]. Ideal wound dressings should (i) be non-toxic, (ii) retain moisture, (iii) protect the wound from friction, (iv) absorb exudate, (v) allow gas/vapor exchange [3,13], and (vi) be easily removable. Hydrogels possess these properties [14], and stimuli-responsive bio-based polymers have, thus, attracted significant attention for diverse biomedical applications [15]. With recent technological advances, hydrogels can be designed as carriers for the timed release of biomolecules or drugs to eliminate overexpressed pathological products, promote vascularisation, and provide antioxidant, anti-inflammatory, and antimicrobial properties to promote wound repair [16,17].

This review describes the recent developments in hydrogels for the treatment of chronic wounds, particularly smart responsive hydrogels that control the release of drugs on demand and eliminate pathological factors by exploiting internal/external stimuli. In particular, we focus on describing design ideas for multiresponsive hydrogels and introduce key functional groups or polymerisation methods to promote the development of future smart medical biomaterials.

## 2. Chronic Wound Characteristics and Hydrogel Design

**Scheme 1:**
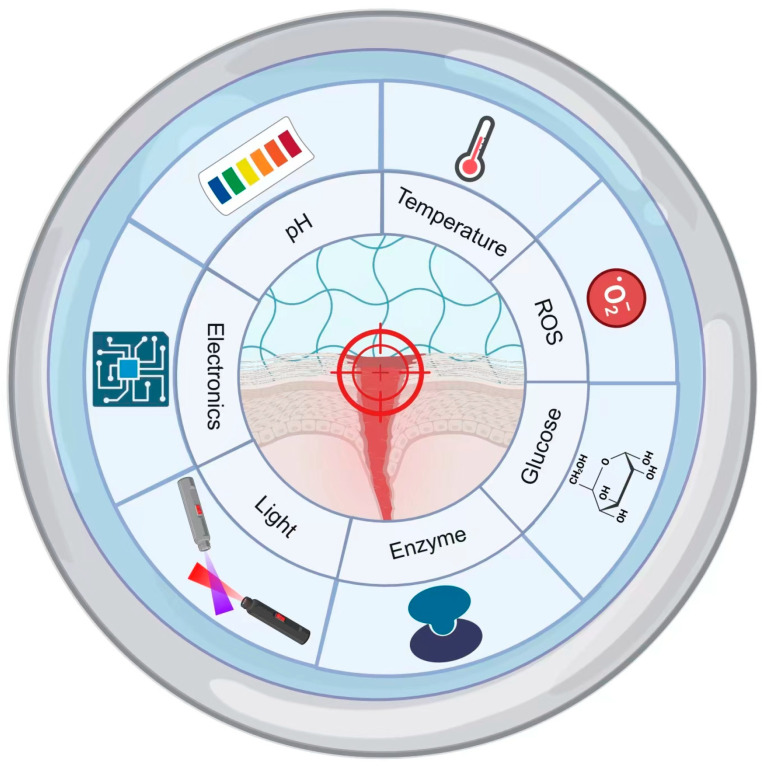
Smart responsive hydrogel wound dressings for chronic wound treatment.

Chronic wound formation as a result of vascular injury, localised pressure, bacterial infection, and uncontrolled inflammation results in a complex wound microenvironment [13]. Traditional wound dressings such as gauze and cotton balls only cover the wound and do not address the underlying problem. Fortunately, the emergence of smart multiresponsive hydrogels in recent years has addressed this problem. Depending on pathological changes such as those in the pH, body temperature, ROS, enzymes, glucose (diabetes), and external stimuli (light, heat, electricity), various different hydrogel designs have been developed, as shown in Scheme 1.

Skin injury and healing are dynamic processes influenced by various factors, and skin wounds have a wide range of pH values [4]. The normal skin surface is slightly acidic, having a pH of 4.0–6.5. However, wounds become alkaline because of injury and the subsequent exposure of internal tissue and body fluids to the external environment [18,19]. Further, if the damage is severe, the wound site can become acidic owing to bacterial infection caused by the lack of an immune barrier, anaerobic respiration, and persistent inflammation resulting from defective capillaries in the wound tissue, and this is particularly common in chronic wounds [4,20,21]. In addition, in wound healing, ROS is a double-edged sword. Inflammatory monocyte activation leads to an increase in pro-inflammatory cytokines and ROS levels. However, the excess production of ROS exacerbates tissue damage [22,23]. Further, the dysregulation of macrophage function in individuals with diabetes leads to a reduced ability to switch to the more reparative M2 phenotype [24], and the high-glucose environment of the wound promotes bacterial growth [25]. Normally, matrix metalloproteinases (MMPs) help remove the damaged extracellular matrix (ECM) and play a role in remodelling. In chronic wounds, the nuclear factor kappa-light-chain-enhancer of activated B cells (NF-κB) is activated depending on the wound severity and ROS levels, and this upregulates MMPs, but excess unregulated MMPs are detrimental, destroying the ECM and preventing wound healing [26]. Together, these factors constitute the unique pathological environment of chronic wounds. Hydrogels are designed to respond dynamically to the characteristics and the pathological products of the wound. They can shrink/swell on demand at the wound site or degrade to release a drug, thus alleviating symptoms and eliminating the pathological products.

Hydrogels that are responsive to external stimuli have several advantages, including controllability and versatility. For example, light energy can be used as a polymerization initiator [27], thereby controlling the mechanical strength of the gel and improving stability [28]. In addition, light can act as a switch for photothermal and photodynamic reactions to achieve wound shape adaptation, drug release, and antimicrobial effects by generating thermal and reactive oxygen radicals [29,30,31]. In addition, damage to the skin induces an endogenous electric field (EF), and previous studies have shown that electrical stimulation has a strong influence on the adhesion, migration, and proliferation of fibroblasts and endothelial cells [32,33]. Thus, endowing a hydrogel with electrical conductivity and applying electrical stimulation to the wound can aid in the healing process. Several groups have developed hydrogel systems with specific targets by integrating these internal factors and manipulating external conditions. They have also made their own choices about how to embed different response switches into hydrogels.

**Figure 1:**
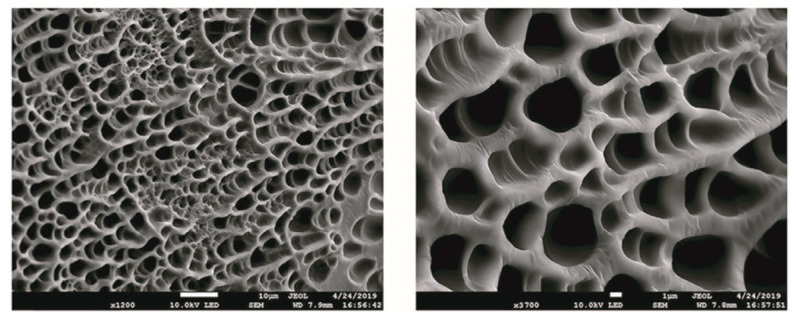
Cross-linked mesh structure of hydrogels observed using scanning electron microscopy [45].

Generally, the chemical modification or the copolymerisation of various monomers are the most commonly used method for synthesising multi-stimuli-sensitive hydrogels [34,35]. By attaching different reactive groups to the monomers or forming functional chemical bonds between them, hydrogels can respond to pathological conditions or external stimuli. Generally, two main processes are employed: physical and chemical crosslinking. Physically cross-linked hydrogels are easier to prepare but have low stability and mechanical strength and are non-permanent. These physical interactions are typically based on hydrogen bonds, ionic chelation, hydrophobic associations, and chain entanglement. In contrast, chemically crosslinked hydrogels are permanent three-dimensional network polymers formed by irreversible covalent bonding and are structurally stable [36], as shown in Figure 1. Several chemical species and reactions are exploited in multiresponsive gels, including polymerisation (including bulk, solution, suspension, and emulsion polymerisation), enzyme-mediated reactions, click chemistry using Schiff bases, Michael addition, etc. [37,38,39,40]. Earlier reviews have discussed synthetic methods for hydrogels in detail, and the reader is directed to these for further information [41,42,43,44].

The design of drug delivery systems aims to achieve controlled drug release in diseased tissue, and the type of stimulus that triggers the release can influence the synthetic method used to prepare responsive hydrogels. For example, hydrogels formed by physical cross-linking through non-covalent interactions are generally less stable and more sensitive to stimuli. Thus, they are suitable for rapid drug release. On the other hand, hydrogels formed by chemical cross-linking are more stable and controllable, making them more favourable for slow-release drugs [46]. For application in complex chronic wound environments, it is increasingly agreed that hydrogels should not be limited to only one type of cross-linking. Rather, hydrogels should release a certain amount of drug in a certain time to eliminate pathological factors, and the selective combination of internal and external stimuli should, thus, achieve a fine-tuned release. In particular, smart responsive hydrogels used in chronic wounds should be composed of biocompatible natural or synthetic polymers; the products of chemical bond breakage or phase transition should be easily eliminated or degraded [47]. To achieve this, different modified polymers and appropriate cross-linking methods have been chosen, and the stage at which drugs, metal ions, or bioactive substances are added has been tailored to achieve the desired effect for smart response therapy.

## 3. Smart Responsive Hydrogel Design

### 3.1. pH-Responsive Gels

#### 3.1.1. pH-Responsive Design

**Figure 2:**
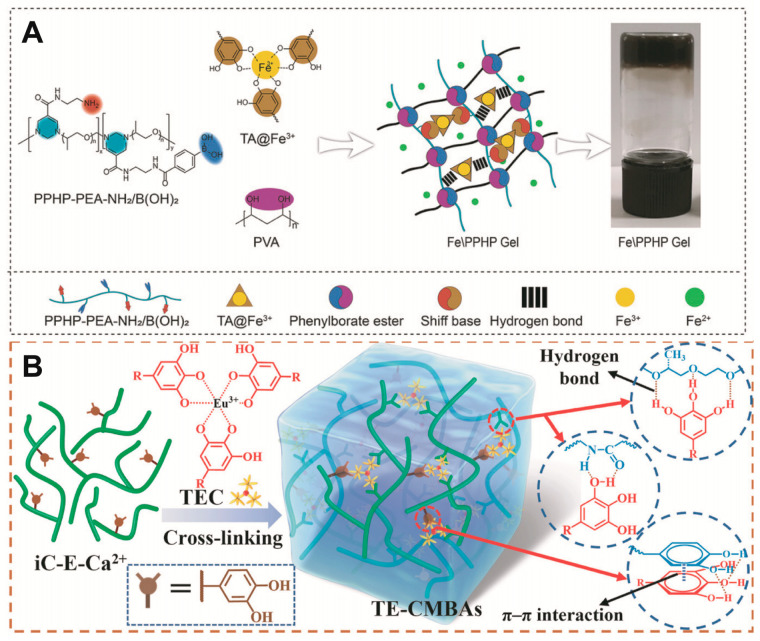
(**A**) Chemical bonding during hydrogel preparation [77]. (**B**) TA–metal ion coordination complex crosslinked citrate-based mussel-inspired bio-adhesives [29]. Reproduced with permission.

Changes in pH are typical of chronic wounds. Therefore, one of the most common types of hydrogels used in chronic wound care are those designed for acidic environments. There are several ways to construct pH-responsive hydrogels. One is to use hydrogels that contain bonds that break within a certain pH range (Figure 2A). Typically, these bonds are stable at physiological pH but hydrolysed under weakly acidic conditions. The second approach is the use of pH-sensitive polymeric materials. Most of these polymeric materials are polyelectrolytes, the p*K*_a_ of which is within the physiological pH range [48]. In addition, pH-responsiveness can be achieved using acid-catalysed hydrolysis [49,50], nanoparticles [51], and electrostatic interactions [45].

#### 3.1.2. Schiff Bases

Schiff bases form reversible imine bonds, which impart smart response properties to hydrogels in acidic environments [52,53]. The flexibility and versatility of Schiff bases make them important for chemical and biological research [54,55]. Further, there is a wide degree of flexibility for the selection of different carbonyl compounds for reaction with different amines [56], and it is relatively easy to introduce amino or aldehyde groups into polymeric compounds via chemical modification [48,57]. Crucially, Schiff bases can coordinate with metal ions via the hybrid orbitals of their nitrogen atoms and lone pairs to form metal complexes with different stabilities and functions [58], and some such ligands and their complexes have shown good antibacterial, anti-inflammatory, antioxidant, and other biological activity [59,60,61].

The structural and functional characteristics of aldehydes and amines are key to the formation of Schiff bases [62]. For example, chitosan (CS) contains amino and hydroxyl groups and provides a rich backbone that can be chemically modified for various purposes [63]. Further, CS is inexpensive and readily available [64,65]. However, modified chitosan has better antibacterial and free radical scavenging ability than pristine CS [66,67,68]. Therefore, CS and its derivatives are often used as monomers in pH-responsive hydrogels. For example, Fang et al. reported a novel multi-functional hydrogel comprising two components. Schiff bases were formed between aldehyde-modified polyethylene glycol (PEG) and quaternised chitosan (QCS) N [66]. Similarly, tunicate cellulose nanocrystals (TCNCs) can be isolated from the mantle of marine animals, and polydopamine (PDA)-coated TCNCs have been used as the reinforcing agent of QCS. Crucially, the quinone group of PDA can interact with the amino group of QCS to form a Schiff base [30]. To achieve better mechanical properties, Hu et al. designed a double-crosslinked hydrogel. Briefly, oxidised dextran-dopamine (OD-DA) was prepared by forming a Schiff base structure between the amino group in DA hydrochloride and the aldehyde group in oxidised dextran. Subsequently, the amino group in the QCS reacts with the aldehyde group in OD-DA via a Schiff base reaction. The hydrogel achieved antimicrobial and angiogenesis-promoting effects after encapsulating Ag-NPs and deferoxamine (DFO) [69]. Similarly, Liu produced hydrogels by modifying CS with 4-formylphenylboronic acid (FPBA) and innovatively combined the pH-responsiveness with an immune response. Specifically, a Schiff base bond was formed between the amino group of CS and the aldehyde group of FPBA. In addition, the presence of *N*-formyl-met-leu-phe (fMLP) recruited a large number of neutrophils to gather at the site of infection, creating a low-pH microenvironment that triggered hydrogel degradation. The Fas ligand then induces apoptosis in activated neutrophils and promotes inflammation resolution. Further, macrophages remove apoptotic neutrophils, and this process activates key signalling pathways to promote the anti-inflammatory phenotypic transformation of macrophages and tissue regeneration [70,71].

Catechol groups are widespread in nature and have been particularly studied in marine mussels, where they act as glues. They can be oxidised or coordinated with metal ions [72]. Notably, the catechol moiety functionalisation of QCS enhances its adhesion for use as a dressing, and the tensile adhesion strength of acid-treated hydrogel adhesives is significantly reduced such that they can be removed on demand [31,73,74]. Cationic amino acids can also promote the destruction of the hydration layer between the catechol and the tissue, thus enhancing its adhesion. For example, l-arginine side chains contain many amino groups that favour the formation of Schiff base bonds [17]. Tannic acid (TA) is rich in catechol units and contains several highly concentrated phenolic groups (Figure 2B). TA can chelate europium ions, and these complexes promote angiogenesis. Specifically, under acidic conditions, the metal–phenol coordination bonds break and release metal ions that facilitate wound healing [29]. In addition, there is no clear indications to suggest that Eu ions is particularly toxic compared to other heavy metals [75,76].

#### 3.1.3. Boronic Ester Bonds

Polymers containing borate bonds are ideal pH-responsive biomaterials [78]. Boronate bonds are formed by the condensation of molecules containing 1,2- or 1,3-diols with boronic acid derivatives [79]. Benzene boric acid (BA) is a Lewis acid with a unionised hydrophobic form and an ionised hydrophilic form in aqueous solution. Notably, ionised BA easily bonds with sodium alginate containing 1,2-diol groups via reversible covalent bonds to form a more hydrophilic structure. When the hydrogel is exposed to an acidic wound, the hydrogel structure is destroyed because of the dissociation of the boronic ester, thus facilitating drug release [80].

#### 3.1.4. p*K_a_*

Usually, pH-responsive hydrogels contain either weakly acidic or basic groups. These groups can accept or release protons at different pH values. Acrylic acid (AA) is a weak acid and undergoes deprotonation at pH values above the dissociation constant of its carboxylic acid group, resulting in hydrogel swelling. Weak polycations contain derivatives of ammonia, such as chitosan, p(l-lysine). Notably, the protonation of nitrogen via its lone pairs provides a positive charge to the macromolecular chain. The consequent charge repulsion causes the hydrogels to swell in acidic media below the p*K_a_* [20,46]. Accordingly, Cui et al. created a hydrogel that actively regulated wound pH; briefly, the hydrogel could release or remove H^+^ ions from different microenvironments to tune the pH of the wound surface precisely and accelerate wound healing [81].

#### 3.1.5. Electrostatic Interactions

Bovine serum albumin (BSA) is a natural protein that can be crosslinked with internal electron-deficient polyesters to form hydrogels via amino-yne click chemistry [82,83,84,85,86]. Crucially, BSA chains are negatively charged in neutral environments, and, under these conditions, basic fibroblast growth factor (bFGF) can bond to the BSA chain segments via electrostatic interactions. In a weakly acidic microenvironment, the charge of BSA is eliminated, resulting in the release of bonded bFGF, which promotes wound healing [45].

The pH plays a key role in physiological processes involved in wound healing [81]. pH-responsive hydrogels not only release drugs at the wound sites but also act as pH regulators. However, pH differences between individuals can affect drug release behaviour and must be considered when designing the hydrogel.

### 3.2. Thermoresponsive Gels

**Figure 3:**
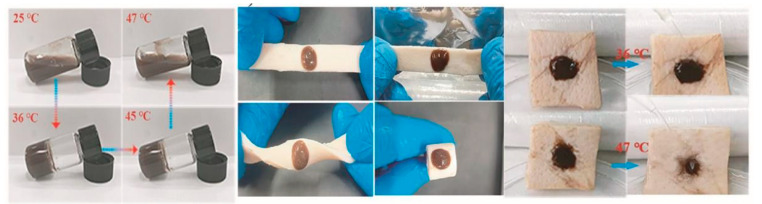
Phase transition of gels at different temperatures and their behaviour on porcine skin [91]. Reproduced with permission.

Materials with a lower critical solution temperature (LCST) between room temperature and physiological temperature have been widely used in biomedical engineering (Figure 3). Notably, such hydrogels solidify when their temperature exceeds the LCST, making them ideal candidates for use in irregular wound dressings [87,88].

*N*-Isopropylacrylamide (NIPAM) is often used for hydrogel preparation [89,90]. For example, Yan et al. incorporated Ag-nanoparticle-modified reduced graphene oxide nanosheets (Ag@rGO) into a polymer containing NIPAM. On phrase transformation into a hydrogel, strong synergistic coordination interactions between the Ag@rGO nanosheets and collapsed PNIPAM chains maintained the hydrogel state and enhanced its stability at low temperatures [36]. However, patients with chronic wounds do not experience significant changes in body temperature; therefore, the temperature stimulus is often used in conjunction with other stimuli when designing hydrogels.

### 3.3. ROS-Responsive Gels

**Figure 4:**
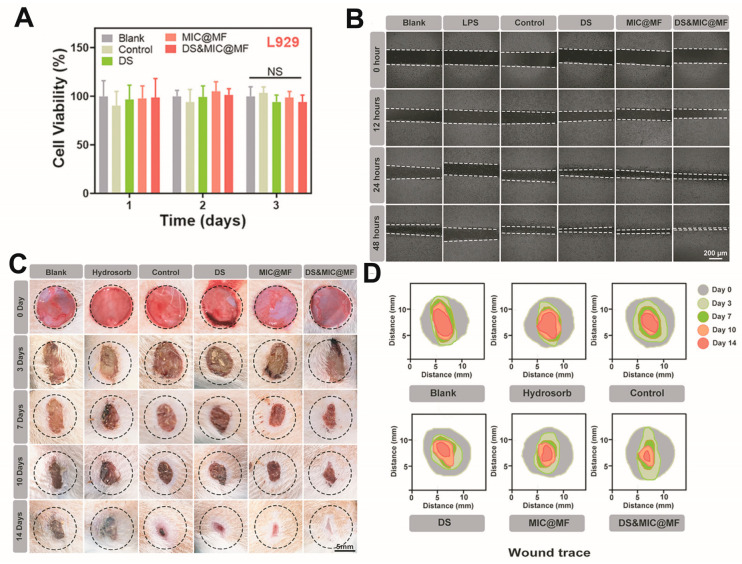
(**A**) Cell viability after incubation with the different hydrogel groups. (**B**) Cell scratch assay, to validate effects on promoting cell growth/migration. (**C**,**D**) In vivo experiments to validate the effect on wound model healing. Reproduced with permission [94].

Excess ROS accumulated in wounds not only induce strong inflammatory responses but can also restrict angiogenesis and hinder wound tissue regeneration [92,93,94,95,96]. The design of ROS-responsive hydrogels is typically based on borate ester bonds and thiol groups. However, an alternative design is the incorporation of ROS-responsive nanoparticles and cross-linking agents. For example, catechol groups can scavenge overproduced ROS and shorten the inflammatory phase [53]. Furthermore, in the presence of ROS, the oxidation of TA to its quinone form can increase the degradation of the hydrogel network, thereby promoting rapid drug release [97]. For example, Li et al. encapsulated copper ions in an ROS-sensitive TA scaffold, and copper-based metal–phenolic networks (MPNs) released the drug to the wound upon an increase in the ROS levels [98]. In addition, Zhao et al. developed an ROS-responsive hydrogel that was cross-linked by the reaction between phenylboronic acid (PBA) and hydroxyl groups. This hydrogel was gradually degraded with an increase in the concentration of H_2_O_2_, demonstrating that such hydrogels have the ability to respond to ROS [93]. Similarly, TA-conjugated nanoparticles (PPBA-TA NPs) with ROS-scavenging and antimicrobial properties were prepared based on the antioxidant effects of TA and ROS-responsive phenylboronic acid ester (PBAE). The PPBA-TA NPs were then reacted with a polyvinyl alcohol (PVA) solution to form injectable hydrogels. This hydrogel acts as an effective ROS-scavenging agent to alleviate inflammation, promote cell migration, and accelerate wound closure [99]. Wu developed a dual-carrying hydrogel that possessed good biodegradability and could achieve the spatio-temporal delivery of diclofenac sodium and Mangifera [94], as shown in Figure 4. It promotes blood vessel proliferation and accelerates wound healing. Guo et al. also developed a hydrogel containing micelles (MIC). The thiol groups present in amphiphilic polymers make the MIC responsive to ROS exposure, which could disrupt the MIC and cause the release of encapsulated paeoniflorin [100].

ROS-responsive hydrogel dressings reduce ROS levels in the wound environment while enabling intelligent drug release. However, as mentioned above, ROS can be a double-edged sword. For example, maintaining ROS at normal levels allows antimicrobial activity to occur without causing a dramatic inflammatory response [101]. This can reduce the use of exogenous antimicrobials, further reducing the risk of side effects.

### 3.4. Glucose-Responsive Gels

Hyperglycaemia is typical in diabetes, and glucose-responsive hydrogels are commonly used to treat diabetic ulcers. Glucose contains an *ortho*-diol group, and PBA forms a more hydrophilic structure when combined with glucose after ionisation, thereby facilitating drug release. In addition, hydrogels doped with glucose oxidase (GOX) and cutanea lectin A can be used for glucose-responsive hydrogel design. Based on this, a glucose-responsive hydrogel microneedle was prepared by the in situ copolymerisation of gelatine methacrylate, the glucose-responsive monomer 4-(2-acrylamide-ethylaminoformyl)-3-fluorobenzene-boric acid (AFPBA), and gluconic insulin (G-insulin). Thus, this hydrogel could control glucose levels by releasing insulin in diabetic patients [102]. Similarly, by grafting PBA onto poly(ethylene glycol) succinate–benzaldehyde (PEGS-BA), the PBA moiety formed a dynamic phenylboronic acid ester with a catechol structure. Thus, the hydrogel released metformin in highly glycaemic environments [17]. Yang used a hydrogel loaded with GOX to break down excess glucose into hydrogen peroxide and glucuronic acid, thereby altering the microenvironment of hyperglycaemic wounds [103]. The hydrogel particles contain complexes of the lectin concanavalin A (ConA) and 2-glucosyloxyethyl methacrylate (GEMA). The GEMA-ConA gel particles selectively recognised glucose, and the swelling ratio of the GEMA-ConA gel particles gradually increased with the increase in glucose concentration. These results indicate that smart gel particles can be used as tools in drug delivery systems to treat diabetes [104].

Diabetic ulcers are typical chronic wounds, and the high glucose concentration in diabetic wounds causes vasoconstriction and inhibits blood vessels, which blocks the supply of O_2_ and impedes the healing process. Further, a high glucose environment can exacerbate bacterial infection [17,105,106]. Therefore, controlling blood glucose levels at the wound site is key in the design of hydrogels for the treatment of diabetic ulcers.

### 3.5. Enzyme-Responsive Gels

Wound-specific enzyme expression can be used to design smart-response hydrogels, for example, the degradation of hyaluronan-based hydrogels that are cross-linked by an ethylenediaminetetraacetic acid (EDTA)−Fe^3+^ complex by bacterial hyaluronidase (HAase). On hydrogel degradation, Fe^3+^ is rapidly absorbed by the surrounding bacteria and subsequently reduced to Fe^2+^, which reacts with H_2_O_2_ to form hydroxyl radicals that damage the proteins and nucleic acids, thus yielding an antibacterial effect [107].

MMPs can also degrade the extracellular matrix, which is involved in tissue remodelling, and the overexpression of MMPs can contribute to the development of chronic wounds. Therefore, exosomes have been loaded into MMP-degradable PEG smart hydrogel, and this has been shown to be beneficial for diabetic wound healing [108].

Enzyme-responsive hydrogels degrade only in the presence of certain enzymes but remain stable in other environments. Therefore, these smart responsive hydrogels are very specific [109]. Crucially, depending on the severity of the wound, the level of enzyme expression varies, and, accordingly, the ability of a hydrogel to release a drug varies, achieving controlled release. If designed as a multi- or cascade response system, they may be favourable for achieving successful wound control.

### 3.6. Photo-Responsive Gels

**Figure 5:**
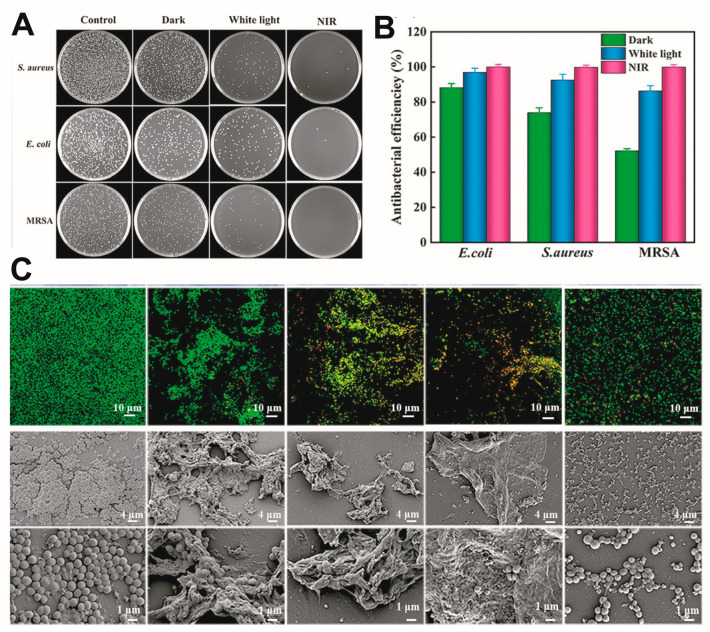
(**A**,**B**) The antibacterial activity of hydrogels against *S. aureus*, *E. coli*, and MRSA. (**C**) Antibacterial capacity displayed with SEM images of biofilms after different treatments. Reproduced with permission [91].

The use of light as a stimulus provides a wealth of inspiration for hydrogel design. Altered polymer conformations or degradation on exposure to light can trigger drug release, and photocurable hydrogels can be used for 3D printing and the development of customised wound dressings [46,110]. In existing studies, photothermal therapy (PTT) and photodynamic therapy (PDT) have been widely used for chronic wound treatment. The combination of these novel therapies with photo-responsive hydrogels not only maintains the time- and position-controlled release of the cargo but has also been adopted for antibacterial treatment. For example, the heat generated by near-infrared (NIR) radiation can be used to achieve physical sterilisation, promote micro-blood circulation, and release drugs or metal ions [111,112,113,114,115]. In addition, the heat generated can be used to induce a phase change in the hydrogel to match the shape of a wound [116]. During PDT, photosensitisers (PSs) can produce cytotoxic ROS at specific wavelengths and induce bacterial death through oxidative stress. However, some PSs can be repelled by negatively charged bacterial cell membranes. To solve this problem, metal–organic frameworks (MOFs), porous materials comprising organic linkers and metal nodes, are often used for PDT [117]. The combined use of PDT and PTT can often yield enhanced results [91]; see Figure 5.

Photo-responsiveness can also be achieved using encapsulated drug carriers. For example, a carrier encapsulating a drug that breaks down under ultraviolet (UV)/NIR irradiation. In one example, Pluronic F127 containing azo groups was prepared, and cyclodextrin (CD) was used to modify QCS because of the inherent photosensitivity of CDs to azo groups. Under 365 nm UV irradiation, micelles loaded with curcumin were released from the hydrogel, yielding antibacterial effects and encouraging wound healing [30]. In another example, on NIR-II light irradiation (1064 nm), the shell of liposomes burst to release an encapsulated drug, thus enhancing the PTT effect for synergistic bacterial elimination [116].

The role of oxygen in wound therapy has long been recognised. Wang et al. developed a NIR-excitation-based device that increases the portability of oxygen therapy. The device consisted of an upper layer for replaceable oxygen generation, a unidirectional delivery system, and a lower layer of perfluorinated hyperbranched polymer/gelatine hydrogel. This hydrogel could be used as an oxygen reservoir for precise delivery to wounds. In contrast, Zhang et al. proposed an interesting material that was not strictly a photo-responsive hydrogel. They used calcium alginate hydrogels loaded with *Weissella* and lipid-membrane-encapsulated *Chlorella vulgaris* to reduce inflammation and the hypoxic microenvironment for chronic wound healing by producing NO and O_2_ in dark and light environments, respectively [118].

Photo-responsive hydrogels offer the advantage of controlling the release behaviour of the hydrogel by adjusting the timing and intensity of light irradiation. Moreover, light irradiation is a non-contact method that requires inexpensive and portable equipment, making it convenient for clinical use. Common light sources for photo-responsive hydrogels are UV and IR light. However, it should be noted that UV light may cause further damage to the fragile wound tissue after long-term exposure, whereas IR light is safer and can penetrate deeper into the tissue [119]. In addition, sunlight is also an important factor to consider in practical applications.

### 3.7. Electro-Responsive Gels

Electroactive hydrogels can be constructed using conductive polymers as monomers. In addition, the introduction of metal nanoparticles, metal ions, or carbon compounds (e.g., graphene) can provide hydrogels with conductivity [120] and confer additional benefits [121,122,123], such as enhanced physical properties and antimicrobial effects. However, the homogeneity of the dispersion of the conductive medium inevitably affects electrical conduction and must be considered when designing such hydrogels.

Tang et al. designed a conductive scaffold by homogeneously dispersing PDA-reduced-graphene oxide (pGO) in a polymer system. The distribution of pGO in the hydrogel provided a channel for the transmission of electrical signals, which affected cell affinity [124]. In addition, Walker et al. recently prepared a biocompatible conductive hydrogel using a choline-based bio-ionic fluid [32]. Moreover, Lei created electroactive hydrogels by crosslinking with dynamic borate ester bonds and hydrogen bonds, which improved the current transmission and facilitated intercellular signalling in the tissue [33]. Furthermore, Jiang et al. developed a dual-conducting (electrical and ionic) hydrogel with a low impedance across a wide frequency range, resulting in more efficient charge injection during stimulation. This ensured efficient signal exchange and energy delivery between the circuits and soft skin tissue [125].

**Figure 6:**
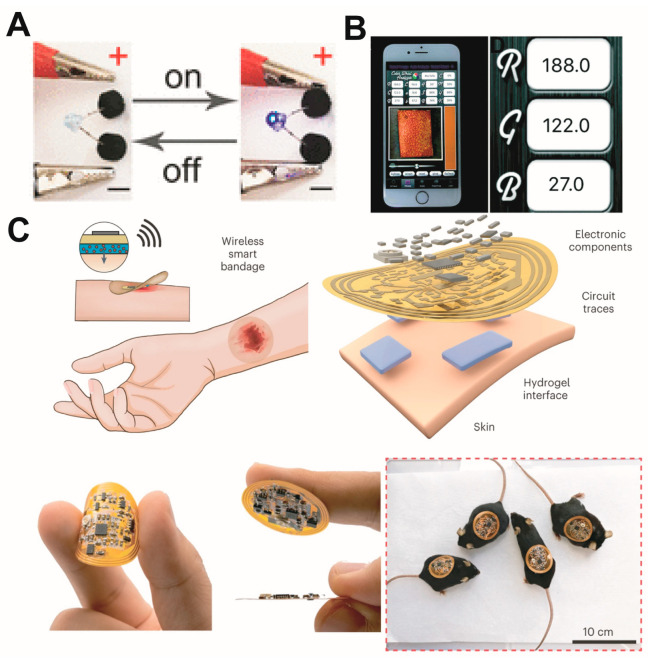
(**A**) Conductivity of hydrogels [124]. (**B**) Intelligent wound monitoring [19]. (**C**) Smart bandage with integrated sensors and stimulators [125]. Reproduced with permission.

Crucially, this hydrogel can conduct electricity, allowing it to be connected to smart electronic devices that can be loaded with modules for various functions. Hydrogel dressings are now evolving into intelligent integrated treatment-monitoring platforms (Figure 6).

In the sections above, we have categorised hydrogels based on their reactions and responsiveness. Next, we will summarise the recent research. In particular, labelled materials, loading, responsive unit, release behaviour and effect will be described. The details are given in Table 1.

## 4. Discussion and Prospects

Chronic wounds are challenging to treat because they involve vascular damage, bacterial infection, and uncontrolled inflammation. These factors lead to abnormal conditions in the wound environment, such as pH imbalance, ROS accumulation, specific enzyme expression, and blood glucose dysregulation. To address these issues, smart hydrogels can be designed to respond to the specific characteristics of chronic wounds and external stimuli. In particular, smart hydrogels can modulate the wound environment by removing pathological factors or delivering drugs in a controlled manner. Moreover, smart hydrogels can also serve as integrated platforms for diagnosis, treatment, and wound healing monitoring. Coupling the action of different structures for achieving multifunctional platforms is the most exciting advance in the field. For example, the development of a light-responsive smart nanoplatform, activated by plasmonic particles, enables on-demand drug release while doubling the amount of drug released [130]. At the same time, the external connection of smart chips or the development of terminal applications can be used for diagnosis and monitoring. Other than this, we know that the inherent properties of hydrogels promote chronic wound healing. Seeking a combination with other therapies can lead to better results [131]. For example, biological therapy [132,133], antibiotics [134], acupuncture [135], etc. The promise of smart response hydrogels for the treatment of chronic wounds is promising. However, designing smart hydrogels for wound healing is not a simple task because it requires the consideration of the complexity and diversity of the human body and the wound conditions. Chronic wounds are vulnerable due to the disruption of the skin barrier and various pathological factors. Therefore, hydrogels must be designed with biocompatibility in mind, including long-term effects and degradation by-products [136,137]. Overly complicated hydrogel designs may not be effective, reliable, or affordable for clinical use. Therefore, research is required to find a balance between the desirable and the practical aspects of smart hydrogel design for wound healing.

## Figures and Tables

**Table 1:** Construction and function of smart responsive hydrogels.

Type of Responsiveness	Materials	Cargo	Responsive Unit	Release Behaviour Study	Effect	Reference
pH-Responsive	Polycaprolactone	Curcumin	Curcumin conjugated effect and reciprocal isomer	**×**	Wound monitoring	[19]
	Carboxymethyl-CS, Sodium alginate	Mn^2+^	MnSiO_3_	**√**	Antibacterial	[51]
	PEG, Acetylenedicarboxylic acid	BSA	Electrostatic interaction	**√**	Proliferation and differentiation of fibroblasts, collagen accumulation, epidermal layer stacking	[45]
	Catechol-QCS, Dibenzaldehyde-PEG	/	Schiff base	**√**	Antibacterial, antioxidant, haemostasis	[53]
	CS quaternary ammonium salt, Oxidised dextran-DA	Ag NPs, DFO	Schiff base	**√**	Antibacterial, angiogenesis	[69]
	FPBA-CS	SiO_2_-Fas Ligand	Schiff base	**√**	Activating immune response	[70]
	Hydroxypropyl acrylate, Acrylic acid, CMCS	Mg^2+^	-COOH, -NH_2_	**×**	Angiogenesis, proliferation and migration of fibroblasts, polarization of macrophages	[81]
	Guar gum, Polyvinyl alcohol	Borax-TA	Borate ester bonds	**√**	Antibacterial, wound monitoring	[126]
ROS-Responsive	Sodium alginate, Pectin, PEG-thioketal	Substance P	Thioketal	**√**	Proliferation of human dermal fibroblasts, ↑inflammation-related genes in macrophages	[15]
	Polyvinyl alcohol, *N*1-(4-boronobenzyl)-*N*3-(4-boronophenyl)-*N*1,*N*1,*N*3,*N*3-tetramethylpropane-1, 3-diaminium (TPA)	Mupirocin, Granulocyte-macrophage Colony-stimulating factor	TPA	**√**	Scavenge ROS, ↓pro-inflammatory cytokines, ↑M2 phenotype macrophages, angiogenesis, production of collagen	[93]
	TA, Polyvinyl alcohol, PBA-modified polyphosphazene	/	Phenylborate ester bond	**×**	Anti-inflammatory, scavenge ROS, antibacterial Re-epithelialization, ↑granulation tissue	[99]
	Sodium hyaluronate, polyvinyl alcohol	MnCoO@PDA/CPH	Catalase-mimic nanozyme	**×**	Anti-inflammatory, re-epithelialization ↑collagen deposition, angiogenesis	[127]
Glucose-Responsive	Gelatine methacrylate, 4-(2-acrylamidoethylcarbamoyl)-3-Fluorophenylboronic acid	G-insulin	Borate ester bonds	**√**	Anti-inflammatory, ↑collagen deposition, glucose control	[102]
	*N*,*N*-Diethylaminoethyl methacrylate, PEG-dimethacrylate, 2-glucosyloxyethyl methacrylate, Modified-ConA	Con A	Reversible cross-link	**×**	Delivery system	[104]
Enzyme-Responsive	Hyaluronic acid	Fe^3+^	Hyaluronidase	**√**	Antibacterial, angiogenesis	[107]
	Four-armed PEG, PEG-sulfhydryl group	Exosome	Matrix metalloproteinase	**√**	Antioxidant, proliferation and migration of cells	[108]
Photo-Responsive	Poly (ethylene glycol) diglycidyl ether, 1,8-octanediamine, 3-perfluorohexyl-1,2-epoxypropane Gelatine	/	PDA	**√**	Improvement of hypoxia	[128]
Electro-Responsive	QCS grafted polyaniline, Oxidised dextran	/	Polyaniline	**×**	Antibacterial, proliferation of cells	[67]
	4-Arm-PEG-Thiol, Carbon nanotubes (CNTs)	Exosome Metformin	CNTs	**√**	Proliferation of cells, angiogenesis	[120]
	PDA-reduced-graphene, oxide (pGO), CS, Silk fibroin	PGO	PGO	**√**	Antioxidant	[124]
Electro-, ROS-Responsive	Polyvinyl alcohol, Human-like collagen, TA	TA, siRNA	Borate ester bond, electric stimulation	**√**	↓ROS and MMP-9, ↑polarization of macrophages, production of collagen, angiogenesis	[33]
Electro-, pH-Responsive	Polyacrylamide, Starch	Drug	-OH, hydrogen bond	**√**	Antibacterial, ↑collagen deposition, angiogenesis, epidermis formation	[49]
Electro-, Thermo-Responsive	QCS grafted polyaniline, Benzaldehyde group-PEG-co-poly(glycerol sebacate)	/	Polyaniline, -NH_2_, quaternary ammonium groups	**×**	Antibacterial, antioxidant	[68]
	Poly-(3,4-thylenedioxythiophene), Polystyrene sulfonate, NIPAM, Acrylamide	/	LCST, ↓interfacial impedance	**×**	Activation of monocyte and macrophage cell, monitoring wound	[125]
pH, ROS-Responsive	Alginic acid sodium salt, HA, 3-aminophenyl boronic acid	Amikacin, Naproxen	Boronic ester bond, micelle	**√**	Antibacterial, anti-inflammatory	[80]
	PBA-grafted oxidised dextran, Caffeic acid-grafted ε-poly-lysine	Mangiferin Diclofenac sodium	Boronic ester bonds, Schiff base, micelle	**√**	Antibacterial, anti-inflammatory antioxidant, angiogenesis	[94]
	HA-PBA, TA	Ag NPs	Boronic ester bond	**√**	Antibacterial, antioxidant	[97]
	Aminated gelatine, Oxidised dextran	ZnO NPs Paeoniflorin	Schiff base Thiol group	**√**	Haemostatic, antibacterial, angiogenic	[100]
pH, Glucose-Responsive	(Dihydrocaffeic acid and l-Arg)-CS, (PBA and benzaldehyde bifunctional PEG)-co-p(glycerol sebacic acid)	Metformin PBA (Reduced-GO)	Schiff base, Phenylboronic ester bond	**√**	Antibacterial antioxidant, haemostasis	[17]
	4,5-Imidazoledicarboxylic acid	Zn^2+^, DFO, Glucose oxidase (GOX)	GOX Metal–organic hydrogels	**√**	Antibacterial, angiogenesis glucose control	[103]
	Hyaluronic acid methacryloyl, Acrylic acid	GOX Copper peroxide	p*K*_a_	**√**	Antibacterial, angiogenic	[129]
pH, Thermo-, Responsive	F127, Oxidative-HA, Poly-ε-l-lysine	Exosome	Schiff base, LCST	**√**	Angiogenesis, re-epithelization, collagen deposition	[6]
	CMC-Na	Diclofenac sodium	-COOH	**√**	Anti-inflammatory	[18]
	NIPAM, AA	Ag NPs	p*K*_a_, LCST	**√**	Antibacterial	[20]
	Hydroxypropyl chitin, TA-Fe^3+^	/	TA-Fe^3+^	**√**	Antibacterial	[72]
Thermo-, Photo-Responsive	F127, Hydroxypropyl methyl cellulose, Cellulose nanofibril	Prussian blue	PTT PDT (Protoporphyrin IX)	**√**	Antibacterial, haemostasis	[91]
	4-Octyl itaconate, PEG, GelMA	Black phosphorus	PTT PDT	**√**	Antibacterial, antioxidant, angiogenesis	[96]
	Fibrinogen, Thrombin	Black phosphorus (BP) Lidocaine	PTT	**√**	Pain relief, antibacterial anti-inflammatory, angiogenesis	[112]
	PLGA PEG PLGA	Niobium carbide	PTT	**×**	ROS-scavenging antibacterial, haemostasis	[113]
	PLGA PEG PLGA	Imipenem@Au liposome	PTT	**√**	Antibacterial, haemostasis anti-inflammatory	[116]
	Alginate, F127	Zn^2+^	PDT (Ce6)	**√**	Antibacterial, collagen deposition, re-epithelialization	[117]
pH, Photo-Responsive	Poly(pentahydropyrimidine), TA-Fe^3+^, Polyvinyl alcohol	/	Schiff base, Phenylborate ester bond, PTT (TA−Fe^3+^)	**√**	Antibacterial, angiogenesis	[77]
	Protocatechualdehyde-Fe^3+^, QCS	/	Catechol−Fe^3+^ Schiff base, PTT	**×**	Antibacterial, haemostatic	[114]
Electro-, ROS, Photo-Responsive	GelMA, [2-(acryloyloxy) ethyl]-Trimethylammonium-chloride (Bio-IL),	Doxycycline	Bio-IL Thioketal	**√**	↑M2 phenotype macrophages, collagen deposition, antioxidant, angiogenesis, re-epithelialization	[32]
pH, Thermo-, Photo-Responsive	TA-Europium	/	Metal–phenolic coordination bonds	**√**	Antibacterial antioxidant, angiogenesis	[29]
	Phenylazo-F127, QCS -graft-cyclo-dextrin, PDA coated tunicate cellulose	Curcumin	Schiff base Host-guest interaction of cyclodextrin and azobenzene	**√**	Antibacterial, haemostasis	[30]
	Gelatine, TA quinone, Borax	/	Boronic ester bond, Schiff base, Polyphenol, PTT (Quinone group)	**√**	Antibacterial, haemostasis	[31]
	Hydroxybutyl-CS, Methacrylic anhydride, Succinic anhydride	/	Succinyl groups Photoinitiator (LAP)	**×**	Delivery systems	[110]
pH, Thermo-, Glucose, Photo-Responsive	HA-PBA, Gelatine, Hyaluronic acid	Metformin @Cu-PDA NPs	Boronic ester bonds PTT	**√**	Antibacterial, anti-inflammatory, angiogenesis, deposition of ECM and collagen	[111]

“**√**” means that the authors conducted a study of release behaviour, “**×**” means that the authors did not design release behaviour experiments; “↑” means to promote or adjust upwards, “↓” means to inhibit or adjust downwards.

## Data Availability

The data presented in this study are available in this article.

## Abbreviations

AA, acrylic acid; ADSC-exo, adipose-derived stem cell-derived exosomes; Ag@rGO, silver-nanoparticle-modified reduced graphene oxide nanosheets; BA, benzene boric acid; BSA, bovine serum albumin; CD, cyclodextrin; ConA, concanavalin A; CS, chitosan; DFO, deferoxamine; EDTA, ethylenediaminetetraacetic acid; EF, electric field; fMLP, N-formyl-met-leu-phe; FPBA, 4-formylphenylboronic acid; GelMA, glycidyl methacrylate; GEMA, 2-glucosyloxyethyl methacrylate; GOX, glucose oxidase; HA, hyaluronic acid; HBC, hyperbranched copolymer; LCST, lower critical solution temperature; MIC, micelles; MMPs, matrix metalloproteinases; MOF, metal–organic framework; MPN, metal–phenolic network; NIPAM, N-isopropylacrylamide; OD-DA, oxidised dextran-dopamine; PBA, phenylboronic acid; PBAE, phenylboronic acid ester; PDA, polydopamine; PEGS-BA, poly(ethylene glycol) succinate-benzaldehyde; PEG, polyethylene glycol; PTT, photothermal therapy; PDT, photodynamic therapy; PSs, photosensitisers; QCS, quaternised chitosan; ROS, reactive oxygen species; TA, tannic acid; TCNCs, tunicate cellulose nanocrystals.
